# Assessing the Safety of Craniotomy for Resection of Primary Central Nervous System Lymphoma: A Nationwide Inpatient Sample Analysis

**DOI:** 10.3389/fneur.2017.00478

**Published:** 2017-09-12

**Authors:** Jonathan Yun, Jingyan Yang, Michael Cloney, Amol Mehta, Suprit Singh, Fabio Massaiti Iwamoto, Alfred I. Neugut, Adam M. Sonabend

**Affiliations:** ^1^Department of Neurological Surgery, Columbia University Medical Center, New York, NY, United States; ^2^Department of Epidemiology, Mailman School of Public Health, New York, NY, United States; ^3^Department of Neurosurgery, Northwestern University Feinberg School of Medicine, Chicago, IL, United States; ^4^Department of Neurology, Division of Neurooncology, Columbia University Medical Center, New York, NY, United States; ^5^Department of Medicine, Division of Hematology and Oncology, Columbia University Medical Center, New York, NY, United States; ^6^Herbert Irving Comprehensive Cancer Center, New York, NY, United States

**Keywords:** cytoreduction, nationwide inpatient sample, primary central nervous system lymphoma, surgery, complications

## Abstract

**Background:**

Unlike many other central nervous system (CNS) tumors, the surgical management of primary central nervous system lymphomas (PCNSL) is traditionally limited by diagnostic biopsy. Studies that predate the use of modern neurosurgical techniques have reported a prohibitive operative morbidity for this surgery. These early experiences have dictated the non-surgical management of PCNSL, whereas resection for cytoreduction is a mainstay of treatment in other CNS malignancies. Recent studies have suggested that craniotomy with the goal of cytoreduction might be associated with a favorable overall and progression-free survival for some patients with PCNSL. To challenge the traditional non-surgical paradigm, it is essential to first investigate the safety of resection for PCNSL.

**Methods:**

To determine the operative morbidity of resection for this disease, we performed a population-based assessment of complications using the nationwide inpatient sample database for the years 1998–2013 for biopsies and open craniotomies for PCNSL and other brain tumors.

**Results:**

Among 95 patients who underwent biopsy and 34 patients who underwent craniotomy, we found no significant difference in complication rates between craniotomy for resection and biopsy procedures for PCNSL (23.16 versus 20.59%). The types of complications differ between diagnoses, with the PCNSL cohort suffering mainly medical complications and the non-PCNSL cohort suffering mainly from neurological complications.

**Conclusion:**

These findings support the safety of craniotomies in PCNSL and help provide a rationale for future prospective studies to evaluate the safety and efficacy of resection for this disease.

## Introduction

Primary central nervous system lymphomas (PCNSL) account for approximately 1–2% of all primary central nervous system (CNS) tumors (1). The standard treatment for these tumors currently consists of chemotherapy with high-dose intravenous methotrexate, as these tumors have demonstrated excellent responsiveness to this initial treatment paradigm (2–4). However, in spite of these treatments, the majority of patients recurs or requires second-line treatments within 1 year. Moreover, many of these patients die, with long-term (5 year) survival in only approximately 15–20% of patients (3, 5, 6). However, unlike many other CNS malignancies, resection for this pathology is discouraged, and surgery is traditionally currently limited to stereotactic biopsy for diagnosis unless mass effect or other reasons for urgent decompression exist (7). Cytoreductive surgery for these lesions has been discouraged as studies from the 1970s to 1990s demonstrated an unacceptable morbidity to the procedure (8–10). Also, sometimes, diffuse distribution and deep brain location of these tumors has raised safety concerns, contributing to the non-surgical treatment paradigm of these tumors (8, 11). However, cytoreductive surgery for PCNSL has not been adequately assessed in the era of modern surgical techniques, such as fluorescein for tumor visualization, MR imaging, neuronavigation, and intraoperative monitoring, which have made resection of brain tumors safer than resection during the time period of the original series. Additionally, the introduction of high-dose methotrexate raises the question of whether the combination of resection followed by this adjuvant regimen might offer a survival advantage for surgical cytoreduction.

Cytoreduction remains the mainstay of surgical management for other CNS malignancies, including gliomas and metastases. Oncologic and survival benefits have been found to be correlated with extent of resection of enhancing lesions, and importantly, these procedures in general are considered safe (12–15). Recent studies have explored the possibility of similar improvements on progression-free survival (PFS) and overall survival (OS) with cytoreductive strategies for PCNSL. Weller et al. investigated the impact of resection using a *post hoc* analysis within the German PCNSL group-1 trial, which was a randomized phase III study involving 526 patients with PCNSL (16). The major benefit of this study was the uniform use of high-dose methotrexate, as the investigators wished to explore the impact of whole-brain radiation therapy in concert with this regimen. The investigators were able to demonstrate that patients who underwent partial or gross total resection had significant PFS and OS benefit compared to patients who underwent biopsy alone. Furthermore, a recent retrospective single institution study performed by Jelicic et al. demonstrated prolonged OS with gross total resection over subtotal resection or biopsy in 27 patients (17). These results suggest a survival benefit with upfront cytoreductive surgical strategies for PCNSL. However, these studies do not investigate the morbidity of resection for this disease. Specifically, no information was presented on the rate of neurologic, regional, and medical complications associated with the craniotomies compared to the commonly performed biopsies. This is important to address, as high complication rates were part of the rationale to avoid gross total resection in early surgical experiences with PCNSL, and while there is an abundance of data to support the safety of surgical resection of other tumors, the data addressing safety specific to PCNSL are sparse (8, 9, 18). In order to best understand the potential beneficial impact of surgery on overall and PFS in PCNSL, a knowledge of complication rates between procedures, namely biopsy and craniotomies, is necessary in order to determine the best options for surgical management and recommendations to the patient.

To investigate the safety of resection for PCNSL, we analyzed the nationwide inpatient sample (NIS), the largest all-payer inpatient database in the United States. Patients were selected for a diagnosis of PCNSL or other primary CNS malignancies. We also identified subjects who underwent a closed/percutaneous biopsy and those who underwent craniotomies. With these data, we compared the complications between craniotomies and biopsies between 1998 and 2013. Also, we investigated the rate and types of complications between surgical procedures for PCNSL versus other CNS pathologies. Last, we explored the factors that contribute to the overall complication risk within the CNS malignancy cohort of the NIS.

## Materials and Methods

### Data Source

The NIS consists of discharge-level data from approximately eight million annual hospitalizations and approximates a 20% stratified sample of community hospitals each year in the United States (19). Each hospitalization in the dataset contains demographic, clinical, and medical resources utilization information. Diagnoses are in International Classification of Diseases, Ninth Revision, Clinical Modification (ICD-9-CM) format, and procedures are in both the Current Procedural Terminology, fourth edition, and healthcare common procedure coding system formats. Because the NIS database is de-identified, the present study did not require Institutional Review Board approval and informed consent.

### Cohort Selection

In the present study, we included patients aged 18 years or older from the NIS between 1998 and 2013 with a principal diagnosis of any of the following: primary malignant neoplasm [(ICD-9) codes 191.1–191.9], brain metastasis [(ICD-9) code 198.3], and primary CNS lymphoma [(ICD-9) code 200.5]. In order to be eligible for the present analysis, the patients needed to have procedures consistent with biopsy or craniotomy according to procedure ICD-9 codes. Furthermore, procedures that can yield cerebrospinal fluid (CSF) pathology, such as spinal tap [(ICD-9) code 03.31], ventriculostomy [(ICD-9) code 02.2], ventriculopuncture [(ICD-9) 01.02], ventricular shunts [(ICD-9) codes 02.31, 02.34, 02.39], and catheter insertion [(ICD-9) codes 01.28, 03.90], were identified for PCNSL patients (Table 1).

**Table 1:** Comparison of complication rates by type of procedure in primary central nervous system lymphomas (PCNSL) (ANOVA test): no difference was found in complication rates (any complication type) between PCNSL patients who received biopsy versus craniotomy.

Complication	Craniotomy *N* (%)	Biopsy *N* (%)	Cerebrospinal fluid diagnosis *N* (%)
Total no. of patients	34	95	347
No	27 (79.41)	73 (76.84)	249 (78.3)
Yes	7 (20.59)	22 (23.16)	98 (28.2)

*p = 0.458*.

### Complications

Complications were categorized into three groups: infectious complications, neurological complications, and medical complications. Neurological complications included iatrogenic cerebrovascular infarction or hemorrhage [(ICD-9) code 997.02], hematoma complicating a procedure [(ICD-9) codes 998.11 and 998.12], intracerebral hemorrhage [(ICD-9) code 431], unspecified intracranial hemorrhage [(ICD-9) code 432.9], and stroke [(ICD-9) code 434.91]. Medical complications included myocardial infarction [(ICD-9) codes 410.00–410.91], acute heart failure [(ICD-9) codes 428.21, 428.23, 428.31, 428.33, 428.41, 428.43], cardiac arrest [(ICD-9) code 427.5], paroxysmal ventricular tachycardia [(ICD-9) code 427.1], pulmonary failure [(ICD-9) codes 518.81, 518.4, 518.5, 518.8], pulmonary embolus [(ICD-9) code 415], precipitous blood loss/transfusion [(ICD-9) code 790.01/V58.2], postoperative shock [(ICD-9) codes 998.00–998.09], non-postoperative shock [(ICD-9) codes 785.50, 785.51, 785.52, 785.59], and acute kidney failure [(ICD-9) code 584]. Infectious complications included meningitis [(ICD-9) codes 320–322.9], surgical site infection [(ICD-9) codes 998.30, 998.31, 998.32, 998.59, 998.83], intracranial abscess [(ICD-9) code 324.0], bacteremia [(ICD-9) code 790.7], septic shock [(ICD-9) codes 785.52, 998.02], and pneumonia [(ICD-9) codes 997.31, 997.32, 481, 482.0–482.9].

### Other Variables

We abstracted demographic and hospital-level characteristics from NIS, including patient age (grouped as patients aged <40, 40–79, and ≥80 years), length of stay (<3, 3–5, and >5 days), race/ethnicity (white, black, Hispanic, and other), type of admission (urgent, elective, and other), source of admission (emergency room, hospital/facility, and other), and hospital bed size (small, medium, large, and unknown). The Charlson Comorbidity Index (CCI), a weighted index based on the 1 year pre-index period and all available diagnosis codes, was used to control for the overall health status of the study population (20). The comorbidity index was stratified numerically as 0, 1, 2, and 3, as a surrogate for the number of comorbid conditions at the time of diagnosis.

### Statistical Analysis

Demographic, clinical, and hospital-level characteristics of the study population were summarized by frequency and percentage. In order to identify risk factors associated with complications following PCNSL treatment, both bivariate and multivariate logistic regression were performed. Odds ratio with 95% confidence interval was reported. A *p*-value <0.05 was used to determine statistical significance. All the statistical analyses (Chi-square and *t*-tests) were carried out using SAS v.9.4 (SAS Institute, Cary, NC, USA).

## Results

We identified 2,348 patients with a diagnosis of PCNSL and 486,638 patients with a diagnosis of other malignant CNS pathologies, including primary malignant lesions (*n* = 141,545) as well as CNS metastases (*n* = 345,093). Of the patients with PCNSL, 129 received a surgery, of whom, 34 (26.4%) underwent a craniotomy, with the remaining 95 (73.6%) receiving a closed/percutaneous biopsy for diagnosis. Within the other pathology cohort, 80,588 patients received surgery, out of which 65,217 (77.6%) underwent a craniotomy, and 15,371 (22.4%) received a biopsy.

Of 2,348 patients assigned a PCNSL ICD-9 diagnosis, only 447 received a procedure that could have yielded a diagnosis of PCNSL (CSF diagnosis = 318, biopsy = 95, craniotomy *n* = 34). This means that 1,901 patients lacked a procedure ICD-9 code that would have yielded diagnostic CNS pathology. To account for this difference, we hoped to identify potential misdiagnoses of PCNSL, such as systemic lymphoma or malignancy. We identified 623 non-CNS diagnostic surgeries, which corresponded with 171 co-diagnoses of systemic lymphoma or malignancy. Even with this, however, 1,278 patients with a PCNSL diagnosis did not have a CNS or non-CNS diagnostic procedure assigned. This is likely due to coding limitations of the NIS dataset.

### Complications after Craniotomy Compared to Biopsy in PCNSL

The complication rates for surgical procedures (biopsy versus craniotomy) that were performed within the cohort of patients with PCNSL were identified and compared. Out of the biopsy cohort (*n* = 95), 22 patients (23.16%) had a complication. This was not significantly different from the group of patients who underwent craniotomy (*n* = 34), of whom, seven patients (20.59%) had a complication (*p* = 0.458). Also, patients who had undergone a procedure in which CSF was obtained, and a diagnosis could have been made on this sample (*n* = 347) had a similar complication rate (28.2%) (Table 2).

**Table 2:** Comparison of complication rates by tumor type in patients who underwent a craniotomy (Chi-squared test): no difference in complication rates (any complication type) was found between patients who had craniotomy for any other tumors versus craniotomy for primary central nervous system lymphomas (PCNSL).

Complication	PCNSL *N* (%)	Other tumor combined *N* (%)
Total no. of patients	34	65,217
No	27 (79.41)	56,846 (87.16)
Yes	7 (20.59)	8,371 (12.84)

*p = 0.194*.

### Other Factors Associated With Risk of Complications

Demographic and procedure-related variables were identified from the NIS in order to determine whether these were associated with an overall risk of complications. Of the factors assessed, older age, extended hospitalization, and belonging to a racial minority group were associated with increased rates of complications. Lower complications were associated with non-urgent admissions, non-emergency room admissions, and increasing hospital size. No association was seen with CCI scores or diagnosis (PCNSL versus other malignancy) when considering all procedures together (Table S1 in Supplementary Material).

### Complications for Craniotomy in PCNSL versus Other CNS Malignancies

Craniotomies are commonly performed on other malignant tumor types, such as primary malignant gliomas and metastases, and generally have an acceptable complication rate as a first-line surgical option. We, therefore, compared complications seen with craniotomies in PCNSL against the standard of craniotomy for other CNS malignancies. We identified 65,217 patients who had undergone craniotomy for non-PCNSL CNS malignancies. Of these, 8,371 (12.84%) had a complication. This was not statistically different from the complication rate seen with craniotomies for PCNSL (20.59%) (*p* = 0.194) (Table 3).

**Table 3:** Comparison of complication rates by tumor type in patients who got biopsy (chi-squared test): patients with primary central nervous system lymphomas (PCNSL) who underwent a biopsy had a higher rate of complications than those who got biopsy for any other tumors.

Complication	PCNSL *N* (%)	Other tumor combined *N* (%)
Total no. of patients	95	15,371
No	73 (76.84)	13,829 (89.97)
Yes	22 (23.16)	1,542 (10.03)

*p < 0.001*.

### Complication Rate after Biopsy for PCNSL versus Biopsy for Other CNS Malignancies

Biopsies accounted for 15,371 (19.4%) of the procedures performed in the non-PCNSL malignancy cohort. This contrasts with the PCNSL cohort, in which most patients received a biopsy (73.6%). Biopsy for PCNSL had a significantly higher complication rate (*n* = 22, 23.16%) than biopsy for other CNS malignancies (*n* = 1,542, 10.03%) (*p* < 0.001) Table 4. In light of this higher complication rate, we sought to investigate the nature of the complications, and whether or not, there was a difference in complication types. Of the 22 biopsied PCNSL patients with a complication, the majority (14.7%) suffered medical complications. This is not different from the types of complications seen in PCNSL patients who underwent a procedure that obtained CSF. In comparison, the majority of complications seen in the biopsy cohort of non-PCNSL malignancies were neurologic in nature (Table 5). The medical complications noted in PCNSL patients were primarily cardiopulmonary events such as myocardial infarction, acute heart failure, cardiac arrest, paroxysmal ventricular tachycardia, pulmonary failure, pulmonary embolus, postoperative shock, non-postoperative shock, and acute kidney failure (Table S2 in Supplementary Material).

**Table 4:** Types of complications by diagnosis (ANOVA): significantly higher rate of complications seen in primary central nervous system lymphomas (PCNSL) cohort versus other tumors, regardless of biopsy or cerebrospinal fluid (CSF) diagnosis.

Type of complication	PCNSL biopsy *N* (%)	Other tumor combined biopsy *N* (%)	PCNSL CSF diagnosis *N* (%)
Nervous system	9 (9.5)	1,039 (6.8)	37 (11.6)
Medical	14 (14.7)	571 (3.7)	34 (10.7)
Infection	9 (9.5)	276 (1.8)	27 (8.5)

*p* < 0.001.

**Table 5:** Factors associated with the risk of complication (regardless of procedure): among the statistically significant predictors of complications were increasing age, extended hospitalization, and race.

Factors	Odds ratio (95% confidence interval)	*p*-Value
**Age, years**		
<40	Ref.	
40–59	1.023 (0.992, 1.055)	0.155
60–79	1.091 (1.058, 1.124)	**<0.001**
≥80	1.220 (1.173, 1.269)	**<0.001**
**Length of stay, days**		
<3	Ref.	
3–5	1.018 (0.992, 1.045)	0.181
>5	2.096 (2.048, 2.145)	**<0.001**
**Race**		
White	Ref.	
Black	1.023 (0.992, 1.054)	0.143
Hispanic	1.061 (1.022, 1.102)	**0.002**
Other	1.045 (1.024, 1.066)	**<0.001**
**Type of admission**		
Urgent	Ref.	
Elective	0.713 (0.691, 0.735)	**<0.001**
Other	0.909 (0.885, 0.933)	**<0.001**
**Source of admission**		
Emergency room	Ref.	
Hospital/facility	0.676 (0.660, 0.693)	**<0.001**
Other	1.040 (1.019, 1.062)	**<0.001**
**Bed size**		
Small	Ref.	
Medium	0.969 (0.938, 1.000)	**0.048**
Large	0.966 (0.939, 0.993)	**0.015**
Unknown	1.184 (1.041, 1.347)	**0.010**
**Comorbidity index**		
0	Ref.	
1	1.240 (1.207, 1.273)	**<0.001**
≥2	1.585 (1.546, 1.625)	**<0.001**
**Diagnosis**		
PCNSL	Ref.	
Other tumors	1.167 (1.018, 1.338)	**0.026**

*Non-urgent admissions, non-emergency room admissions, and larger volume hospitals were associated with lower rates of complications (multivariable logistic regression). Bolded *p*-values indicate statistical significance*.

We performed an additional multivariable analysis to explore the increased rate of medical complications observed in patients with PCNSL. We adjusted for demographic factors that were independently associated with complications such as age, length of hospitalization, and race (Table 2). After adjusting for these variables, we detected no statistically significant difference in the rate of medical complications between the PCNSL cohort and non-PCNSL cohort, suggesting these demographic characteristics may in fact play a role in the increased rate of medical complications (Table S3 in Supplementary Material).

## Discussion

Previously published high complication rates associated with craniotomy for PCNSL (8, 9) have contributed to the wide acceptance of diagnostic biopsy as the first-line surgical option followed by high-dose methotrexate, with or without radiation (7). The role of craniotomy for surgical resection of PCNSL in the era of modern neurosurgical techniques requires a fresh mindset, as most data on the safety and efficacy of resection precede modern neurosurgical techniques and high-dose systemic methotrexate, and thus might be outdated (21). Recently, a few studies demonstrated a potentially beneficial impact on survival with cytoreduction in PCNSL (16, 17). An important aspect that is lacking from this body of research is a comprehensive understanding of the complication rates, which is necessary to evaluate the risk-benefit ratio that might justify resection. In this study, we described a population-based assessment of complication rates of patients undergoing craniotomy versus biopsy for PCNSL compared to other intracranial tumor pathologies.

Our analysis using the NIS revealed no significant difference in complication rates between craniotomy and biopsy (20.59 versus 23.16%, *p* = 0.458) for PCNSL. Our complication rate of 20.59% for craniotomies in PCNSL, of which 9.5% were neurologic, demonstrates a stark difference to previously published rates of complications in surgical resection of PCNSL, such as one notable study that demonstrated a 40% permanent neurologic complication rate in patients undergoing craniotomy for resection of PCNSL (8). Our findings suggest that on a national level, the overall safety of performing a craniotomy on a patient with PCNSL is similar to that of performing a biopsy.

Craniotomy for resection of CNS malignancies, such as gliomas and brain metastases, is considered part of the standard of care and is associated with a survival benefit for these patients (22, 23). In such cases, the risk associated with resection is generally accepted. In our study, we did not find significant differences in the complication rates following craniotomy for PCNSL compared to craniotomy for other CNS malignancies. To rule out the possibility of a small sample size leading to a lack of significance, we performed a power analysis to determine if low sample size contributed to the lack of significance observed. To detect a significant difference between a 40% complication rate for craniotomy in PCNSL (consistent with historical data) versus the 12.84% complication rate for craniotomy in other lesions, we would need a sample size of 24 patients. Given that we identified 34 patients with PCNSL who received a craniotomy, it is unlikely that a small sample size alone could explain the lack of significant differences in the complication rates for resection of PCNSL versus other CNS malignancies.

We found a higher rate of complications for biopsy in the case of PCNSL than for the same procedure on other CNS malignancies. The majority of the complications associated with biopsies in PCNSL were medical in nature (14.7%), in contrast to the higher neurologic complication rate seen in the cohort of biopsied other CNS malignancy patients (6.8%, Table 5). Moreover, the complication rate for PCNSL patients who underwent biopsy was similar to the complication rate for patients with PCNSL who did not undergo a craniotomy or a biopsy. One possible interpretation of these findings is that the PCNSL cohort fundamentally represents a unique patient population with different surgical risk factors versus non-PCNSL tumor patients, reflected by the increased medical complication rate among those who received biopsies. After adjusting for race, length of hospitalization, age, and other patient characteristics, we found no statistically significant difference in the rates of complications between the PCNSL cohort and the non-PCNSL cohort, supporting our hypothesis that there may be demographic differences between the two cohorts that explain the increased rate of medical complications that we initially observed (Table S3 in Supplementary Material).

An interesting finding was the increased rate of complications found in patients with CSF diagnosis procedures (28.2%) compared to patients with biopsy (23.16%) and craniotomy (20.59%). We speculate that this may be because the patients who required a CSF diagnostic procedure such as spinal tap, ventriculostomy, ventricular puncture, etc., were likely a baseline sicker population. Future prospective studies would be able to define the characteristics associated with complication rates that could affect the CSF diagnosis population.

The NIS offers a few inherent benefits, as it allows for a better estimation of the true incidence of complications at a national level compared to single institution or even multi-center studies. Data from the NIS have been well validated and has a widely accepted level of accuracy (24). Nevertheless, there are important limitations to consider when using and interpreting these data from the NIS. First, we make the assumption that a craniotomy has the intent of resection for cytoreduction. This assumption may be flawed, as in a small number of cases, a craniotomy may have been performed for the purpose of biopsy rather than for debulking. This is also an important limitation that should be addressed in future prospective trials, as extent of resection can impact OS and neurological outcomes. Other limitations of this study and the use of NIS relate to the ICD-9 coding system, as it can lead to inaccurate identification of procedures as well as diagnoses. This can be seen in this study with the small proportion of PCNSL patients assigned a relevant procedure ICD-9 code (as discussed in the Section “Results”).

In addition, the NIS does not provide details related to the procedure or its complications. For example, there may be a crossover of patients who initially received a biopsy, but eventually required a craniotomy; this nuance would not adequately be captured by the NIS. Also lacking from this dataset is the permanence of complications, specifically neurologic complications that may possibly improve or resolve on follow-up evaluation. Therefore, to understand the safety of craniotomy for PCNSL using modern surgical techniques, institutional series might complement our study, as these could offer more granularity and thus information on the transience or permanence of complications. The NIS also offers no information on the location of the tumor, a parameter that is relevant especially with regards to PCNSL, as these tumors often develop in eloquent areas, or areas that are difficult to access (i.e., deep brain structures), thus increasing the risk of postoperative complications (8, 11, 21). Additionally, the dataset does not provide certain specifications about the patients (clinical history, neurological status, general status, etc.) which may serve as confounders when comparing the PCNSL cohort to the non-PCNSL cohort. Finally, as is the case with all brain tumors, determining the resectability of the tumor in PCNSL is a crucial step in the decision making process and should be part of the consideration when deciding between craniotomy versus biopsy (25).

Our findings suggest that craniotomies performed for PCNSL may be a safe alternative to the current standard of biopsy alone. However, given the limitations found in this dataset, we feel this study should be used as a rationale for future prospective assessments of craniotomy for resection as first-line surgical management for PCNSL.

## Conclusion

In light of recent studies that suggest improved survival with surgical resection for PCNSL, a better understanding of the risk of complications associated with pursuing resection is necessary. In this study, we compared complication rates between craniotomies and biopsies in a cohort of patients with a diagnosis of PCNSL or other malignant brain lesions. We found that no difference in complication rates exist between either procedure in patients with PCNSL. Further, patients who receive craniotomies for PCNSL have similar complication rates to those who receive craniotomies for other brain lesions. Given its limitations, this study cannot definitively assert the safety of craniotomy compared to biopsy. However, it serves as a valuable data point to provide a rationale for further prospective investigations into the comparative safety of biopsy versus craniotomy in patients diagnosed with PCNSL.

## Author Contributions

All authors were involved with gathering the dataset, writing of the paper, interpreting the data, and directing the intellectual trajectory of the project.

## Conflict of Interest Statement

AN is a consultant for Pfizer, Otsuka Pharmaceuticals, Teva Pharmaceuticals, and United Biosource Corporation. He is a member of the Medical Advisory Board of EHE, Intl. FI is a consultant for Merck, Novocure, Abbvie, Regeneron. He receives research support from Merck, Novocure, Celldex, Northwest Biotherapeutics, Bristol Meyers Squibb. All other authors declare no conflicts of interest.
