# Supplementary material for: Assessing the Safety of Craniotomy for Resection of Primary Central Nervous System Lymphoma: A Nationwide Inpatient Sample Analysis
Source: Front Neurol. 2017 Sep 12;8:478. doi: 10.3389/fneur.2017.00478 (PMC5600910; doi:10.3389/fneur.2017.00478)
Supplement: Supplementary file 1 [file Table_1.DOCX]

**Supplemental Table 1:** Procedure ICD-9 codes included in study

| Stereotactic needle biopsy | |
| --- | --- |
| 01.11 | Closed [percutaneous] [needle] biopsy of cerebral meninges |
| 01.13 | Closed [percutaneous] [needle] biopsy of brain |
| 01.14 | Open biopsy of brain |
|  |  |
| Craniotomy |  |
| 01.24 | Other craniotomy |
| 01.25 | Other craniectomy |
| 01.31 | Incision of cerebral meninges |
| 01.39 | Other incision of brain |
| 01.53 | Lobectomy of brain |
| 01.59 | Other excision or destruction of lesion or tissue of brain |
|  |  |
| CSF |  |
| 03.31 | Spinal tap |
| 02.2 | Ventriculostomy |
| 01.02 | Ventriculopuncture through previously implanted catheter |
| 02.34 | Ventricular shunt to abdominal cavity and organs |
| 01.28 | Placement of intracerebral catheter(s) via burr hole(s) |
| 03.90 | Insertion of catheter into spinal canal for infusion of therapeutic or palliative substances |
| 02.39 | Ventricular shunt to extracranial site NEC |
| 02.31 | Ventricular shunt to structure in head and neck |
