# Supplementary material for: Assessing the Safety of Craniotomy for Resection of Primary Central Nervous System Lymphoma: A Nationwide Inpatient Sample Analysis
Source: Front Neurol. 2017 Sep 12;8:478. doi: 10.3389/fneur.2017.00478 (PMC5600910; doi:10.3389/fneur.2017.00478)
Supplement: Supplementary file 2 [file Table_2.DOCX]

**Supplementary Table 2:** Subtypes of cardiopulmonary complications seen in patients with PCNSL and other CNS tumors that underwent biopsy.

|  | PCNSL N (%) | Other tumor combined N (%) |
| --- | --- | --- |
| **Cardiac** | 8 (40.0) | 159 (25.9) |
| Ventricular tachycardia | 3 (15.0) | 43 (7.0) |
| MI | 0 (0) | 55 (9.0) |
| Cardiac Arrest | 0 (0) | 28 (4.6) |
| Heart Failure | 1 (5.0) | 2 (0.3) |
| Blood Loss/shock | 4 (20.0) | 31 (5.0) |
| **Pulmonary** | 12 (60.0) | 455 (74.1) |
| Acute Respiratory Failure | 11 (55.0) | 310 (50.5) |
| Acute Lung Edema | 0 (0) | 4 (0.7) |
| Pulmonary Insufficiency | 1 (5.0) | 141 (23.0) |
