# Supplementary material for: Assessing the Safety of Craniotomy for Resection of Primary Central Nervous System Lymphoma: A Nationwide Inpatient Sample Analysis
Source: Front Neurol. 2017 Sep 12;8:478. doi: 10.3389/fneur.2017.00478 (PMC5600910; doi:10.3389/fneur.2017.00478)
Supplement: Supplementary file 3 [file Table_3.DOCX]

**Supplementary Table 3:** Multivariable analysis of association of diagnosis (PCNSL vs. Non-PCNSL) and medical complications

| Covariates | Odds ratio | 95% CI | p-value |
| --- | --- | --- | --- |
| Diagnosis |  |  |  |
| PCNSL | Ref |  |  |
| Non-PCNSL | 1.328 | 0.846-2.086 | 0.217 |
| Age, yr |  |  |  |
| <40 | Ref |  |  |
| 40-79 | 1.262 | 1.182-1.348 | <0.001 |
| ≥80 | 1.222 | 1.121-1.332 | <0.001 |
| Gender |  |  |  |
| Males | Ref |  |  |
| Females | 0.857 | 0.828-0.886 | <0.001 |
| Race/ethnicity |  |  |  |
| White | Ref |  |  |
| Black | 1.171 | 1.114-1.231 | <0.001 |
| Hispanic | 1.182 | 1.101-1.270 | <0.001 |
| Other | 1.268 | 1.168-1.377 | <0.001 |
| Length of stay, days |  |  |  |
| <3 | Ref |  |  |
| 3-5 | 0.700 | 0.663-0.738 | <0.001 |
| >5 | 1.471 | 1.406-1.539 | <0.001 |
| Type of admission |  |  |  |
| Emergency | Ref |  |  |
| Urgent | 0.703 | 0.666-0.741 | <0.001 |
| Other | 0.510 | 0.477-0.545 | <0.001 |
| Source of admission |  |  |  |
| Emergency room | Ref |  |  |
| Hospital/facility | 0.989 | 0.924-1.058 | 0.746 |
| Other | 0.711 | 0.675-0.748 | <0.001 |
| Hospital bed size |  |  |  |
| Small | Ref |  |  |
| Medium | 1.048 | 0.984-1.117 | 0.143 |
| Large | 0.958 | 0.905-1.014 | 0.135 |
| Charlson comorbidity index |  |  |  |
| 0 | Ref |  |  |
| 1 | 1.377 | 1.302-1.457 | <0.001 |
| ≥2 | 1.720 | 1.632-1.812 | <0.001 |
